# A CRISPR-Cas9 System for Genome Editing of *Fusarium proliferatum*

**DOI:** 10.1038/s41598-019-56270-9

**Published:** 2019-12-27

**Authors:** Massimo Ferrara, Miriam Haidukowski, Antonio F. Logrieco, John F. Leslie, Giuseppina Mulè

**Affiliations:** 1 Institute of Sciences of Food Production, National Research Council, Bari, Italy; 2 Department of Plant Pathology, Kansas State University, Manhattan, Kansas USA; 3 Institute of Biomembranes, Bioenergetics and Molecular Biotechnologies, National Research Council, Bari, Italy

**Keywords:** Fungi, Molecular biology

## Abstract

*Fusarium proliferatum* causes diverse diseases of many economically important plants. The fungus produces several mycotoxins of which the fumonisins are the most toxic. Currently, deletion of key genes for mycotoxin biosynthesis is a laborious and time-consuming procedure. We developed a novel CRISPR/Cas9-based genome-editing tool for the direct delivery of preassembled Cas9 ribonucleoproteins into protoplasts of *F. proliferatum*. Our CRISPR–Cas9 system couples a site-specific double-strand DNA break mediated by two Cas9 ribonucleoproteins with microhomology recombination requiring only 50-bp regions flanking the target gene. This system reduces the risk of off-target mutations and minimizes the risk of altering any gene adjacent to the target region. We used this tool to delete a polyketide synthase gene (*FUM1*) required for fumonisin biosynthesis. The mutants generated are no longer able to produce fumonisins, confirming the key role of *FUM1* in fumonisin biosynthesis. Our CRISPR-Cas9 system is an important new tool for genetic studies of *Fusarium*.

## Introduction

*Fusarium proliferatum* is a globally distributed fungal pathogen that can attack diverse crops, including wheat, maize, rice, asparagus, date palm, garlic, onion, ornamental palms, and Chinese chive^1^. *Fusarium proliferatum* produces multiple mycotoxins, including fumonisins, moniliformin, beauvericin, fusaproliferin and fusaric acid^2^. The fumonisins are the most important of these toxins, not only for the mycotoxicoses with which they are associated, which includes esophageal cancer and neural tube defects in humans, cancer in rats, pulmonary edema in swine and leukoencephalomalacia in horses^3^, but also for their regulation in the international grain trade. There are four major classes of fumonisins – A, B, C and G – with the B-type the most toxic and the most commonly recovered. Fumonisin B_1_ (FB_1_) is categorized as a group 2B carcinogen by the International Agency for Research on Cancer^4^, and is potentially carcinogenic to humans.

The 16-gene fumonisin biosynthetic gene cluster (*FUM*) is known in multiple fumonisin-producing fungal species^5–8^, and includes genes that encode biosynthetic enzymes, regulatory factors and transport proteins^9^. Gene inactivation and/or heterologous expression studies^10–14^ have identified the functions of most of the genes in the *FUM* cluster. *FUM1* encodes a polyketide synthase responsible for synthesis of the linear polyketide that forms the chemical backbone common to all fumonisins. The *FUM* cluster in *F. proliferatum* is collinear with the *FUM* clusters of *Fusarium verticillioides* and *Fusarium oxysporum*^8,15,16^. The *F. proliferatum FUM1* sequence is 85% identical to the *F. verticillioides* homologue^15^. In *F. verticillioides*^7,17^, the *FUM1* gene is required for fumonisin biosynthesis. The essential role of *FUM1* in the biosynthesis of fumonisins by *F. proliferatum* has been demonstrated^18^.

The Clustered Regularly Interspaced Short Palindromic Repeats (CRISPR) system is a recently described genome-editing tool for gene knockout, gene insertion and gene replacement. The most popular type II CRISPR systems have two components: (i) a CRISPR-associated Cas9 endonuclease from *Streptococcus pyrogenes*, and (ii) a single-guide RNA (sgRNA), which is the fusion of a precursor CRISPR RNA (precrRNA) and a trans-activating CRISPR RNA (tracrRNA)^19^. The sgRNA recognizes the target DNA sequence and forms a DNA/RNA duplex at the recognition site. The Cas9endonuclease then cleaves this DNA/RNA duplex. The resulting double-strand DNA breaks are repaired by non-homologous end-joining, or by homology directed repair (HDR), if a donor DNA repair template is co-transformed into cell. The HDR repair system is particularly effective for complete gene knock-outs when coupled with dual *in-vitro-*assembled Cas9 ribonucleoproteins (RNPs) that target the 5ʹ and the 3ʹends of the gene coding region.

Type II CRISPR-Cas9 systems also have been used in other filamentous fungi including: *Aspergillus nidulans* and *Aspergillus aculeatus*^20^, *Aspergillus oryzae*^21^, *Aspergillus fumigatus*^22–24^, *Aspergillus niger*^25^, *Aspergillus carbonarius*^26^, *Trichoderma reesei*^27^, *Neurospora crassa*^28^, *Alternaria alternata*^29^, *Penicillium chrysogenum*^30^, *F. oxysporum*^31^, and *Fusarium graminearum*^32^. However, no application has been reported for *F. proliferatum* or other members of the *Fusarium fujikuroi* species complex.

In this study, we used a Type II CRISPR-Cas9 system to inactivate a key gene in the fumonisin biosynthetic cluster in *F. proliferatum*. Our working hypotheses were: (i) *in-vitro*-assembled RNPs could be used with a donor DNA repair template for direct co-transformation of *F. proliferatum*, and (ii) the *FUM1* gene was essential for the synthesis of fumonisin. This work advances the field by providing a new, simple tool for making knockout mutants in members of the *F. fujikuroi* species complex, and by confirming the essential role of *FUM1* in fumonisin biosynthesis in *F. proliferatum*.

## Results

### Designing sgRNA protospacers for deleting *FUM1*

Our goal was to develop a Cas9-mediated gene deletion system in *F. proliferatum*. We assembled *in vitro* dual Cas9 RNPs and a DNA repair template flanked by microhomology regions adjacent to the target gene.

**Figure 1:**
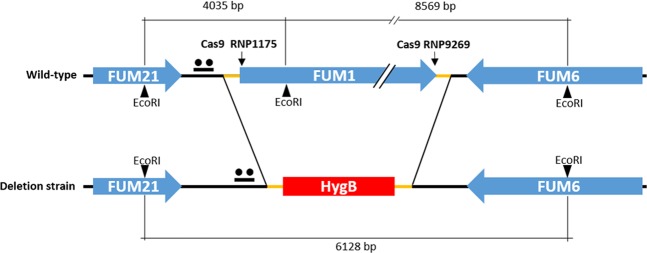
Schematic representation of the *FUM1* gene deletion by *in vitro*-assembled dual Cas9 ribonucleoproteins coupled with homology directed repair (HDR). The cleavage sites of the *in vitro*-assembled Cas9 RNP1175 and RNP9269 (↓), the 50 bp microhomology regions for HDR (orange segment), the *Eco*RI cut sites, and the pks*FUM1*-specific probe (●●) are represented for the genomic locus of the wild-type ITEM 7595 and *ΔFUM*1 deletion strain.

To delete *FUM1*, we used two sgRNAs (Table 1) that directed Cas9 RNP DNA binding and cleavage to two specific locations. To reduce the possibility of deleting non-*FUM1* coding sequences, sgRNAs nearest the start and stop codons were chosen. Based on predicted PAM sites and sgRNA scores, two sgRNAs were selected: (i) sgRNA1175, which was 15 bp downstream of the *FUM1* start codon, and (ii) sgRNA9269 which was 11 bp upstream of the *FUM1* stop codon (Fig. 1). This process leaves 26 (15 + 11) nucleotides of the *FUM1* coding region flanking the *HygB* insertion. *In silico* analysis of off-target mutations by the selected sgRNAs confirmed the specificity of the two protospacers. Both of the selected sgRNAs had <15-bp off-target identity with other genomic regions in *F. proliferatum*.

**Table 1:** sgRNAs used in this study

ID	Protospacer sequence 5′→3′	PAM site
sgRNA1175	TCACCCCCGAGTACCGCTGT	AGG
sgRNA9269	TGATGCGTATCTGGAAATGA	AGG

### Cas9-mediated *FUM1* deletion in *F. proliferatum*

Cas9-mediated cleavage of the *FUM1* gene and the HDR of the selectable marker (*HygB*) were coupled through microhomology recombination with dual RNPs directly into the cleavage sites. Microhomology recombination occurs in the 50-bp regions homologous to the nucleotide sequences adjacent to the sgRNA1175 and sgRNA9269 cleavage sites (Fig. 1).

**Figure 2:**
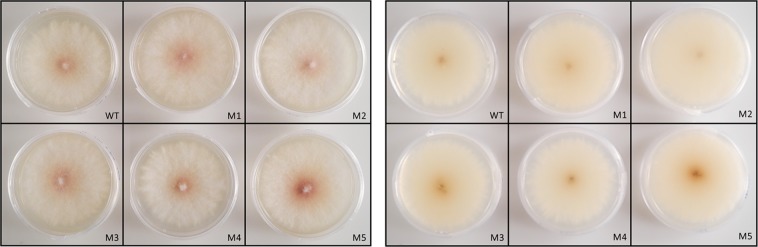
Colony morphology and pigmentation of *F. proliferatum ∆FUM1* mutants obtained with the *in-vitro*-assembled RNPs coupled with HDR method. Front and reverse sides of the colonies are shown in the left and right panels, respectively. WT = wild-type strain ITEM 7595; M1-M5 = five arbitrarily selected *F. proliferatum* ∆*FUM1* mutants. The phenotypes were evaluated after 7 days of incubation on PDA at 25 °C under white light (12 h light/dark).

The HDR-*HygB* repair template, which contains the *HygB* expression cassette, was successfully fused to the 50-bp flanking regions by PCR amplification and sequenced (GenBank accession no. MN226410). Following co-transformation of the dual RNPs and the HDR-*HygB* repair template, protoplasts were regenerated and mutants selected on PDA + hygromycin. Eleven putative *∆FUM1* transformants were obtained. Colony morphology and pigmentation of the *F. proliferatum ∆FUM1* mutants were evaluated after 7 days of incubation at 25 °C on PDA. No significant differences were observed between the wild-type and mutant cultures in morphology or pigmentation under the tested growth conditions (Fig. 2).

### PCR screening of *F. proliferatum ∆FUM1* mutants

**Figure 3:**
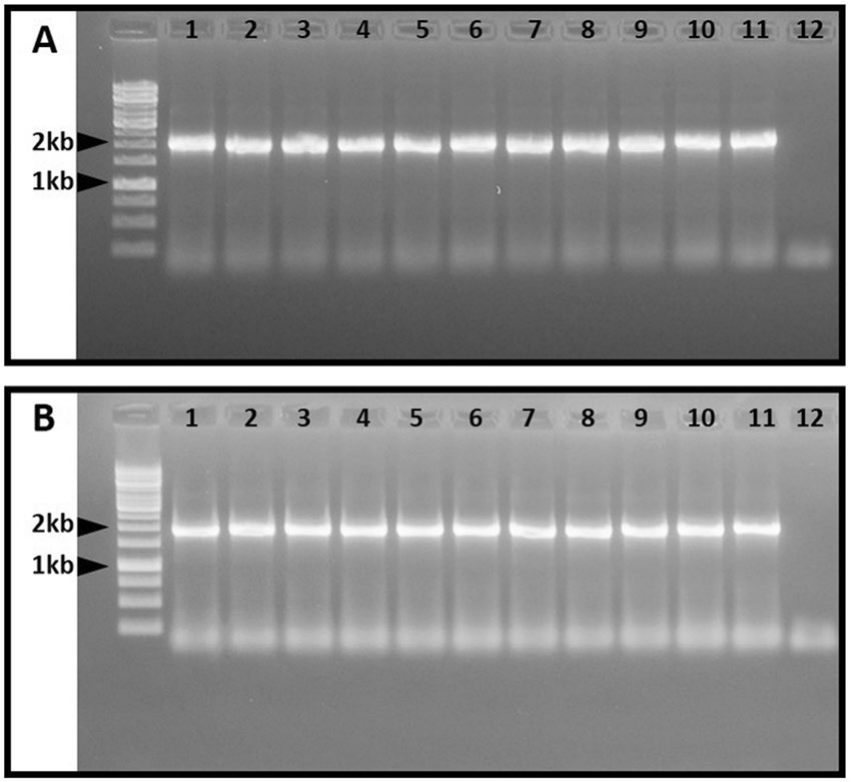
PCR amplification of HDR-*HygB* repair template integration locus spanning the cleavage sites of the *in-vitro*-assembled RNP complexes. Expected amplicon size for the RNP1175 (A) and RNP9269 (B) complexes was 1734 bp and 1619 bp, respectively. M = 1 kb ladder; 1–11 = *F. proliferatum ∆FUM1* mutants resistant to hygromycin B; 12 = wild-type strain ITEM 7595.

The putative *∆FUM1* mutants were screened by PCR amplification of a genomic sequence including both the upstream and the downstream cleavage sites in the RNPs. PCR amplification was performed on DNA from purified cultures of *HygB* resistant *∆FUM1* mutants. The resulting fragments were consistent with integration of the *HygB* cassette into the predicted cleavage sites in all 11 mutants (Fig. 3).

### Southern blot analysis

**Figure 4:**
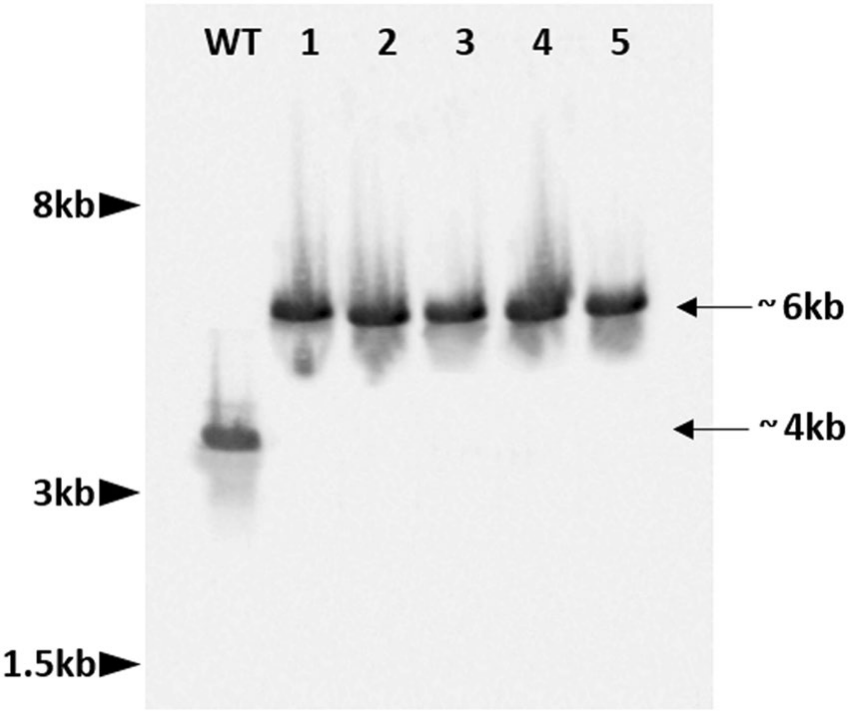
Southern blot of DNA from wild-type strain ITEM 7595 and five arbitrarily selected *∆FUM1* mutants following digestion of genomic DNA with *Eco*RI and hybridization with a *FUM1*-specific probe. In the wild type ITEM 7595 DNA (WT), a genomic fragment of ~4 kb was detected. Lanes 1–5 all contain an ~6 kb DNA fragment predicted from the *FUM1* deletion pattern and HDR of the *HygB* cassette.

To confirm the correct integration of the HDR-*HygB* repair template cassette by CRISPR-Cas9 mediated homologous recombination into the targeted gene locus, a Southern blot analysis was performed with five arbitrarily selected, purified *∆FUM1* transformants. Following *Eco*RI digestion of wild-type genomic DNA and hybridization with a *FUM1*-specific probe (Fig. 4), a 4035 bp genomic DNA fragment (located at position 64909–68942 on scaffold 50 of genome reference strain NRRL62905) was detected. This DNA fragment included portions of the *FUM21* (810 bp) and *FUM1* (2056 bp) genes. *Eco*RI digestion of DNA from all of the *∆FUM1* mutants resulted in a 6128 bp genomic segment. The DNA fragment from the Δ*FUM1* mutants contains the *HygB* cassette (1619 bp) instead of most of the *FUM1* gene and also includes a portion of the *FUM6* gene (2160 bp) (Figs. 1 and 4).

### Fumonisin analysis

The polyketide synthase gene, *FUM1*, was the reporter target gene of this study. The encoded polyketide synthase catalyzes the synthesis of the linear polyketide backbone of the fumonisins. Deletion of *FUM1* in *F. proliferatum* results in mutant strains that are resistant to hygromycin B and are unable to produce fumonisins, as expected.

**Figure 5:**
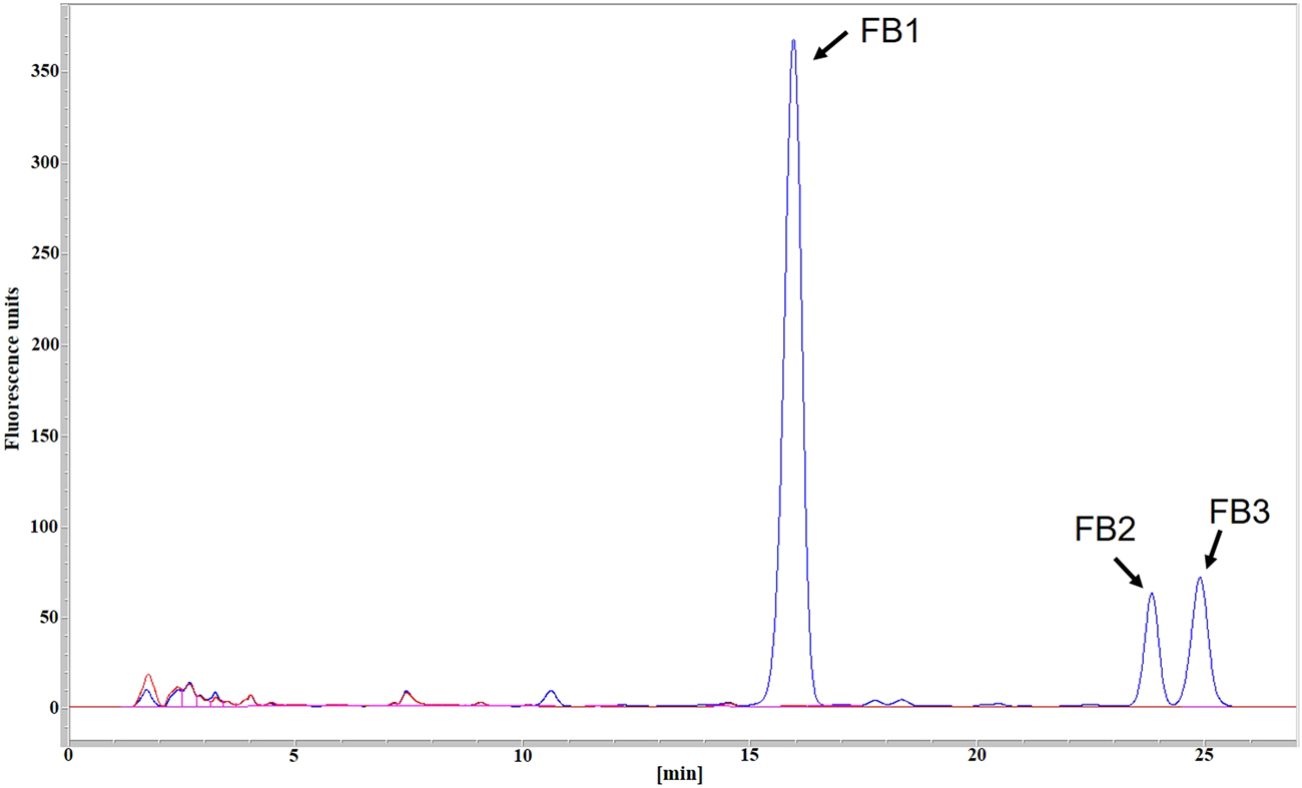
HPLC-FLD chromatograms of culture extracts of wild-type *F. proliferatum* strain ITEM 7595 (blue line) and one arbitrarily selected ∆*FUM1* mutant strain (red line). Retention times: FB_1_, 16.250 min; FB_2_, 23.785 min; FB_3_, 24.968.

We confirmed the role of *FUM1* in fumonisin biosynthesis by analyzing cultures of wild-type strain ITEM 7595 and the *∆FUM1* mutants for the presence of FB_1_, fumonisin B_2_ (FB_2_) and fumonisin B_3_ (FB_3_) by HPLC/FLD. The amounts of FB_1_, FB_2_ and FB_3_ in cultures of ITEM 7595 after 19 days of growth were 272 µg/g, 78 µg/g and 88 µg/g, respectively. None of the *∆FUM1* mutants grown under the same conditions produced detectable FB_1_, FB_2_ or FB_3_ (Fig. 5).

## Discussion

The CRISPR/Cas9 system is an attractive, powerful tool that may replace the classical gene knockout approach based on homologous recombination in filamentous fungi. We describe the application of CRISPR/Cas9 tools to genome editing of *F. proliferatum*. This technique significantly expands the research tools available for *F. proliferatum* and related species in the *F. fujikuroi* species complex.

The tool we developed enables more comprehensive analysis and exploitation of gene function, especially of genes with roles in secondary metabolite biosynthesis. Genes coding for polyketide synthases (*pks*) have previously been used as reporter genes to assess the efficacy of the CRISPR-Cas9 genome editing system in other fungi, *e.g*., *A. fumigatus*^33^ and *F. oxysporum*^31^. Our study confirmed the key role of *FUM1* in fumonisin biosynthesis by *F. proliferatum*. This report is the first of using a CRISPR-Cas9 system to delete a *pks* gene in a mycotoxin biosynthetic pathway. Given the length of the genomic region we successfully deleted (about 8000 bp), our study confirms that the CRISPR-Cas9 system can be used to target multiple genomic loci and/or large genomic regions at a single physical location with a single transformation event.

The method is a simple, near-universal system for gene deletion and has potentially widespread applicability in filamentous ascomycetes. In addition, the size of the genomic region being deleted is theoretically much larger with our system than with the traditional gene deletion approach.

Currently, deletion of genes involved in mycotoxin biosynthesis requires laborious and time-consuming construction of DNA fragments containing selectable markers with large (~750 to 1,500 bp) flanking homology regions^17,34–36^. The CRISPR–Cas9 system we used couples a dual site-specific DNA break, mediated by two *in-vitro*-assembled RNP complexes, with microhomology recombination dependent only on 50-bp regions flanking the targeted deletion site. A similar approach has been used in *F. oxysporum*^31^, but the *HygB* resistance cassette was flanked by ~600 bp homologous sequence both upstream to and downstream of the cleavage sites. In the current study, we demonstrated that 50 bp flanking regions are sufficient for specific recombination at a targeted genomic locus in *F. proliferatum*. The use of very short flanking regions for microhomology recombination offers two additional advantages: (i) it minimizes the risk of altering adjacent gene sequences; (ii) it allows targeting of a gene(s) that is closely spaced in a genomic region. The latter advantage makes this system particularly useful for studying genes located in a cluster that enables the biosynthesis of a secondary metabolite, where very tight targeting may be required to disrupt the function of just a single gene. Indeed, the possibility of deleting multiple clustered genes with a single transformation event could greatly improve the study of their interaction in the biosynthetic process.

The use of microhomology-site direct recombination coupled with Cas9-mediated cleavage was effective with a 60-bp flanking region in *P. chrysogenum*^30^, and with 50-bp or 35-bp flanking regions in *A. fumigatus*.^45^ The success of these approaches requires careful *a priori* studies of nucleotide sequences flanking the target genomic locus in order to avoid sequence homology with other genomic regions. The ability to target a specific genomic region by fusing only a 50-bp flank to the target is particularly important in fungi with significant amounts of DNA duplication, as it reduces the number of unwanted off-target recombination events and multiple site integrations by the HDR-repair template cassette.

The direct transformation of fungal protoplasts with *in vitro*-assembled RNPs also avoids the need to express the Cas9 protein in the fungal host cell and to assemble specific expression cassettes for each sgRNA. Usually both the Cas9 and the sgRNA expression cassettes are included in the same expression vector with a selectable marker that confers resistance to an antifungal agent. This vector is transformed into the fungal protoplasts for either transient expression^20^ or chromosomal integration^32^. Vector-based systems usually are laborious and time-consuming to assemble since they require the construction of multiple vectors, each with different sgRNAs for the different target genes. Cas9 expression usually is controlled by robust, constitutive promoters that guarantee high levels of active protein in the host cell. A significant drawback to high intracellular levels of Cas9 protein is off-target cleavage by the protein and unexpected, and often difficult to identify, genome alterations.

We used a commercially available Cas9 protein for *in vitro* assembly of RNP complexes with the appropriate sgRNAs. A similar approach has been used in other filamentous fungi, such as *T. reesei*^27^, *P. chrysogenum*^30^, *A. fumigatus*^23^, *A. niger*^25^, and *F. oxysporum*^31^. The efficiency of Cas9-mediated cleavage is linked to its nuclear localization, and to its binding to the targeted DNA regions. The nuclear import of the protein results from the fusion of a nuclear localization sequence (NLS) to the Cas9 protein. The commercial Cas9 protein used in this study was imported effectively into the *F. proliferatum* nucleus and cleaved its target site as expected. Indeed, the direct transformation of fungal protoplasts with *in vitro*-assembled RNPs has a much lower risk for off-target mutations than if Cas9 protein expression was mediated by vector transformation. The lowered risk of off-target mutations results from the limited time in which an active form of the Cas9 protein is present in the fungal nucleus. In this study, all eleven of the analyzed mutants had only a single integration event and the expected deletion profile. However, a genome-wide analysis of the *∆FUM1* mutants is needed to confirm our expectation that the genomic changes in the transformants are limited to the changes we report at *FUM1*. The lack of significant morphological differences between the wild-type strain and the *∆FUM1* mutants (Fig. 2) supports this expectation. These results differ from those reported by Sun *et al*.^18^, who found that deletion of *FUM1* and *FUM19* in their *F. proliferatum* strain altered growth rate and conidiation. Indeed, the potential influence of *FUM1* and *FUM19* on fungal growth and conidiation warrants further consideration with these characters evaluated in multiple mutants constructed in different genetic backgrounds that are grown on several media under a number of culture conditions.

Our process enables a strategy wherein RNPs are rapidly assembled *in vitro* with sgRNAs targeting multiple genomic loci. This capability is an important step forward for studies in *F. proliferatum* and other filamentous fungi, as multiple genes in the same metabolic pathway can potentially be deleted simultaneously.

## Conclusion

We developed and applied for the first time in *F. proliferatum* a gene deletion process based on a CRISPR Cas9 Type II system that uses *in vitro* assembled RNPs and a donor DNA repair template. The use of *in-vitro-*assembled RNPs results in only transient exposure of the cells to Cas9, thereby minimizing off-target events due to rapid degradation of the RNPs. A protocol that combines sgRNAs synthesized *in vitro* with a commercially available Cas9 protein may be the most rapid and versatile option for deleting genomic regions in filamentous fungi, and could become the new standard for genetic studies with these organisms.

The ability to potentially target multiple genomic loci, both linked and unlinked, in a single transformation event is a powerful tool to study the relatedness of physically or functionally related genes. These genes might be clustered in a single chromosome region or dispersed throughout the genome. These properties are particularly relevant for studies of biosynthetic gene clusters involved in fungal secondary metabolism, and in the study of multi-copy genes that may have a role in plant pathogenicity. The phenotypes with these multi-locus genetic bases are of theoretical interest and practical importance and have been difficult to study with knock-out technology that permits only one-gene-at-a-time type studies.

## Methods

### Strains and preparation of conidia

We used a wild-type *F. proliferatum* strain, ITEM 7595, from the Agri-Food Toxigenic Fungi Culture Collection (Bari, Italy – http://server.ispa.cnr.it/ITEM/Collection/). The strain was routinely cultured on potato dextrose agar (PDA–Oxoid Ltd., Basingstoke, Hampshire, United Kingdom). The knock-out *∆pks FUM1* strains were grown on PDA supplemented with 100 mg/L hygromycin B (Thermo-Fisher Scientific, Waltham, MA, USA). Conidia suspensions were made from colonies grown on PDA for 7 days at 25 °C. The surface of the colony was scraped with a spatula, and mycelia and conidia were suspended in 10 mL of sterile distilled water. The suspension was filtered through Miracloth (Merck, Darmstadt, Germany). Spores in the resulting suspension were counted with a haemacytometer and the suspension diluted to a final concentration of 10^7^ conidia/mL.

### *sgRNA* design, synthesis and *in vitro* assembly of Cas9-gRNAs

The sgRNAs for *FUM1* (GenBank: KU180047.1) deletion were designed with the Eukaryotic Pathogen CRISPR guide RNA/DNA Design Tool – EuPaGDT (grna.ctegd.uga.edu). The annotated sequence (NCBI accession number GCA_900029915.1) of reference strain NRRL62905 of *F. proliferatum* was used to design the sgRNAs. Parameters guiding design of the sgRNAs (Table 1) used for gene deletion were: (i) sgRNA cleavage sites were near the 5′ or the 3ʹ end of the target gene; (ii) the protospacer adjacent motif (PAM) sequence was (N)20NGG. The potential for off-target cleavage at other loci was checked by BLASTN of the *F. proliferatum* NRRL62905 genome sequence. For *in vitro* transcription, the sgRNA forward primer was fused to a T7 promoter. The sgRNA DNA templates were PCR-assembled and transcribed *in vitro* with the GeneArt Precision gRNA Synthesis kit (Thermo-Fisher Scientific), according to the manufacturer’s instructions. The sgRNA1175 and sgRNA9269DNA templates were assembled by using primers IVT-F-gRNA1175/IVT-R-gRNA1175 and IVT-F-gRNA9269/IVT-R-gRNA9269 (Table S1), respectively. Following *in vitro* transcription, the sgRNAs were purified and quantified with a Qubit RNA BR Assay kit (Thermo-Fisher Scientific). Purified sgRNAs were stored at −80 °C.

The RNP complexes were assembled *in vitro* by using TrueCut Cas9 Protein v2 (Thermo-Fisher Scientific). The RNP complexes were assembled in a final volume of 15 μl by combining in 1 × NEBuffer 3.1 (New England BioLabs, Ipswich, MA, USA), 500 nM of Cas9 enzyme and 500 nM of sgRNA1175 or sgRNA9269. The mixtures were incubated at 25 °C for 15 min to generate RNP complexes designatedRNP1175 or RNP9269, and used immediately to transform protoplasts of *F. proliferatum* (see below).

### Construction of the HDR-*HygB* repair template

The HDR-*HygB* repair template, containing the hygromycin B resistance cassette (*HygB*) from plasmid pYTK079, was constructed by fusing the *HygB* cassette (1619 bp) to 50 bp sequences in NRRL 62905 that flank the upstream and downstream RNP cleavage sites. The *HygB* cassette was PCR amplified from pYTK079 with Platinum Super Fi PCR Master Mix (Invitrogen, Carlsbad, CA, USA) and 500 nM of primers HDR5ʹharmHygB_F and HDR3ʹharmHygB_R (Table S1). PCR amplification conditions were: 98 °C for 30 sec, 30 cycles of 98 °C for 10 sec, 63 °C for 15 sec, and 72 °C for 1 min, followed by a single 72 °C incubation for 5 min for final extension. PCR amplification products were checked following electrophoresis through a 1% agarose gel stained with 1 × GelRed (Biotium, Fremont, CA, USA). The resulting DNA fragment (1719 bp) was purified by using the GeneJET gel extraction kit (Thermo-Fisher Scientific) and cloned into the pCR-XL-2-TOPO vector with the TOPO XL-2 Complete PCR cloning kit (Invitrogen), according to the manufacturer’s instructions. Positive clones were sequenced and the resulting plasmid designated p*HygR*-*FUM1*. The HDR-*HygB* repair template for microhomology recombination was PCR amplified from plasmid p*HygR*-*FUM1* with primers HR5ʹharm*HygB*_F and HR3ʹharm*HygB*_R. PCR amplification products were purified with the GeneJET PCR Purification Kit and checked following electrophoresis through a 1% agarose gel stained with 1 × GelRed.

### Protoplast preparation and transformation

Protoplast preparation and transformation followed that of Coleman *et al*.^37^, with some modifications. Briefly, conidia of ITEM 7595 were inoculated into 100 mL of potato dextrose broth (PDB– Oxoid Ltd., Basingstoke, Hampshire, UK) at a final concentration of 10^4^ conidia/mL and cultivated for 16–18 h at 28 °C with shaking at 250 rpm. Mycelia were harvested by filtration, washed with sterile 0.7 M NaCl, and resuspended in 1.2 M KCl protoplasting buffer (pH 6.5) containing 100 mg/mL of VinoTastePro (Novozyme, Bagsvaerd, Denmark) lytic enzyme mix. The mixture was incubated at 30 °C with gentle shaking (80 rpm) until protoplasts were released (3–4 hours). Protoplasts were separated from mycelial debris by filtering through Miracloth and then pelleted by centrifugation at 4 °C for 15 min at 2360 × *g*. Protoplasts were washed with SuTC (20% sucrose, 50 mM Tris-HCl, pH 7.0, 50 mM CaCl_2_), centrifuged at 4 °C for 15 min at 2360 × *g*, and finally resuspended in SuTC at a concentration of 10^7^ protoplasts/mL. Protoplasts (200 μl, 2 × 10^6^ protoplasts) were co-transformed with RNP1175 and RNP9269 (500 ng each) and 3 μg of purified HDR-*HygB* repair template, and then incubated without shaking at 25 °C for 20 min. Three transformation aliquots were prepared. One milliliter of PSuTC (20% sucrose, 50 mM Tris·HCl, pH 7.0, 50 mM CaCl_2_, 60% polyethylene glycol-3,350) was added to a transformation aliquot and the incubation continued at room temperature for another 20 minutes. The transformation aliquot was transferred to a 15 mL sterile tube containing 3 mL of TB3 medium (20% sucrose, 1% glucose, 0.3% yeast extract, 0.3% Cas-amino acids) and incubated on a rotary shaker (160 rpm) for 18–24 h at 25 °C. Regenerated fungal protoplasts were mixed with molten (45 °C) PDA containing 100 mg/L of hygromycin B, poured into sterile Petri dishes, and incubated at 25 °C for 5–8 days until hygromycin-resistant colonies appeared. Putative transformants were transferred to PDA containing 100 mg/L of hygromycin B. Purified mutant strains originated from colonies subcultured as single microconidia on selective Spezieller Nährstoffarmer agar (SNA) medium (0.2 g/L sucrose, 0.2 g/L glucose, 1.0 g/L KNO_3_, 1.0 g/L KH_2_PO_4_, 0.5 g/L MgSO_4_·7H_2_O, 0.5 g/L KCl, and 15 g/L agar) supplemented with 100 mg/L of hygromycin. Purified *F. proliferatum ∆FUM1* mutants were maintained on selective PDA for further molecular analysis and stored at −80 °C as conidial suspensions in sterile 15% glycerol. Phenotypes of *F. proliferatum ∆FUM1* mutants were evaluated on PDA plates inoculated with a drop (2 μL) of a conidial suspension (10^4^ conidia/mL) in the center of the plate. Inoculated plates were incubated at 25 °C and inspected visually after 7 days.

### PCR analysis of putative *F. proliferatum ∆FUM1* mutants

Genomic DNA of wild-type ITEM 7595 and purified cultures of *F. proliferatum* ∆*FUM1* mutants was extracted by using the GeneJET Plant Genomic DNA Purification Kit. Following quantification with a NanoDrop ND-1000 spectrophotometer (Thermo-Fisher Scientific) and an integrity check following electrophoresis through a 0.8% agarose gel, 10 ng of genomic DNA from each isolate was used for PCR amplification of the HDR recombination sites. A 1734-bp fragment beginning 115 bp upstream of the RNP1175 cleavage site (83 bp upstream of the *FUM1* start codon), which includes the *HygB* cassette (1619 bp), was amplified with primers FUM1_F(−83up) and HygB_R (Table S1). Similarly, a 1744-bp fragment beginning 125 bp downstream of the RNP9269 cleavage site (114 bp downstream of the *FUM1* stop codon), which includes the *HygB* cassette (1619 bp), was PCR amplified with primers HygB_F and FUM1_R(+114dw) (Table S1). PCR amplifications were made with Platinum SuperFi PCR Master Mix (Invitrogen) and 500 nM of each primer. PCR amplification conditions were: 98 °C for 30 sec, then 30 cycles of 98 °C for 10 sec, 63 °C for 15 sec, and 72 °C for 90 sec, and a final extension at 72 °C for 5 min. Amplification products were evaluated following electrophoresis through a 1% agarose gel stained with 1 × GelRed. Both fragments were purified with the GeneJET gel extraction kit. Purified DNA fragments were sequenced by using Sanger sequencing and primers FUM1_F(−83up) or FUM1_R(+114dw).

### Southern blot analysis

A *FUM1*-specific probe was amplified from genomic DNA of ITEM 7595 by using Platinum SuperFi PCR Master Mix (Invitrogen) with 500 nM each of primers probe5ʹ_koFUM1_F and probe3ʹ_koFUM1_R (Table S1). PCR amplification conditions were: 98 °C for 30 sec, 30 cycles of 98 °C for 10 sec, 63 °C for 15 sec, and 72 °C for 1 min, followed by a final extension at 72 °C for 5 min. PCR amplification products were evaluated following electrophoresis on a 1% agarose gel stained with 1 × GelRed. The 591-bp PCR fragment was biotinylated with the PCR DIG Probe Synthesis kit (Sigma-Aldrich, St. Louis, MO, USA), according to the manufacturer’s instructions. Genomic DNA of five arbitrarily selected purified putative *∆FUM1* transformants was extracted by using the GeneJET Plant Genomic DNA Purification Kit. Following quantification with a NanoDrop ND-1000 spectrophotometer) and an integrity check following 0.8% agarose gel electrophoresis, genomic DNAs were digested with *Eco*RI overnight at 37 °C. Fragments separated on a 1% agarose gel and then transferred to a BrightStar-Plus Positively Charged Nylon Membrane (Thermo-Fisher Scientific). The membrane was hybridized with the *FUM1*-specific biotinylated probe and developed with the North2South chemiluminescent hybridization and detection kit (Thermo-Fisher Scientific). Chemiluminescent detection was performed with a ChemiDoc MP Imaging System (Bio-Rad, Hercules, CA, USA).

### Fumonisin extraction and HPLC analysis

The wild type strain (ITEM 7595) and the *∆FUM1* mutants were inoculated on PDA in Petri dishes and incubated at 25 °C. After 19 days the contents of the Petri dishes were collected and the fumonisins extracted. Fumonisins were analyzed with a slightly modified version of the AOAC Official Method 2001.04^38^, which includes an immunoaffinity (IMA) column clean-up of extracts and toxin determination by HPLC/FLD of fumonisins derivatized with *o*-phtaldialdehyde (OPA). Briefly, ~10 grams of the colonized PDA substrate was extracted with 40 mL methanol/water (70:30, v/v) by shaking for 1 h at room temperature. The contents of the flask were filtered through Whatman no. 4 filter paper, and a 10 mL aliquot of the filtrate was mixed with 40 mL of distilled water (50 mL total volume). Ten milliliters of the diluted sample was passed through a FumoniTest^TM^ Wide Bore IMA column (Vicam, Milford, MA, USA). The IMA column was washed with 10 mL PBS (10 mM phosphate buffer, 2.7 mM potassium chloride and 137 mM sodium chloride, pH 7.4). Fumonisins were eluted from the IMA columns with 2 mL of methanol followed by 2 mL of distilled water. Eluted extracts were evaporated under an air stream at 50 °C, dissolved in 1 mL acetonitrile/water (30:70, v/v), and analyzed by HPLC.

The HPLC/FLD determination and confirmation of fumonisins B_1_, B_2_ and B_3_ was performed as previously described^39^. Extracts were derivatized with OPA (1:1, v/v) and mixed for 50 sec in an Agilent 1100 HPLC auto sampler equipped with a binary pump, and the column thermostat set at 30 °C. Three minutes after adding the OPA reagent, 100 μL of the extract was injected by full loop. The analytical column was a Symmetry Shield RP18 150 × 4.6 mm, 5 μm (Waters) with a guard column inlet filter (0.5 μm × 3 mm diam., Rheodyne Europe GmbH Bensheim, Germany). The mobile phase was a binary gradient applied as: (i) 57% of solvent A (water/acetic acid, 99:1, v/v) and 43% of solvent B (acetonitrile/acetic acid, 99:1, v/v) for 5 min; (ii) solvent B was increased linearly to 54% at 21 min, and to 58% at 25 min, and (iii) 42% solvent A and 58% solvent B for 5 min. The flow rate of the mobile phase was 0.8 mL/min. The fluorometric detector was set at wavelengths, ex = 335 nm, em = 440 nm. The retention time for FB_1_, was about 17 min, about 24 min for FB_2_, and 25.5 min for FB_3_.

For fumonisin analyses, a 5 μg/mL FB_1_ and FB_2_ standard stock solution was prepared in acetonitrile/water (1:1, v/v) by dilution of a certified Biopure calibrant solution (Romer Labs Diagnostic, Tulln, Austria). FB_1_ and FB_2_ were measured by calculating peak areas and comparing the results with calibration curves prepared in acetonitrile/water (30:70, v/v) over the range 0.005–5.000 μg/mL. FB_3_ was measured by using the FB_2_ calibration curve. The limit of detection (LOD) of the analytical method was 30 μg/kg for each fumonisin.

## Supplementary information

Supplementary Information
