# Supplementary material for: A CRISPR-Cas9 System for Genome Editing of Fusarium proliferatum
Source: Sci Rep. 2019 Dec 27;9:19836. doi: 10.1038/s41598-019-56270-9 (PMC6934662; doi:10.1038/s41598-019-56270-9)

Supplementary Information

A CRISPR-Cas9 System for Genome Editing of *Fusarium proliferatum*

Massimo Ferrara, Miriam Haidukowski, Antonio F. Logrieco, John F. Leslie, Giuseppina Mulè

Supplementary Table

Table S1. Primers used in this study. Microhomology arms for Homology Directed Repair (HDR) are underlined. Primer sequences specific for amplification of the *HygB* cassette are in bold and nucleotide positions relative to the *FUM1* start[(1)] or stop [(2)] codon are indicated.

Primer ID	Nucleotide sequence 5′→3′	Purpose
IVT-F-gRNA1175<br>IVT-R-gRNA1175	TAATACGACTCACTATAGTCACCCCCGAGTACCG<br>TTCTAGCTCTAAAACACAGCGGTACTCGGGGGTG	PCR assembly of sgRNA1175 DNA template
IVT-F-gRNA9269<br>IVT-R-gRNA9269	TAATACGACTCACTATAGTGATGCGTATCTGGAA<br>TTCTAGCTCTAAAACTCATTTCCAGATACGCATC	PCR assembly of gRNA9269 DNA template
HDR5′harmHygB_F<br>HDR3′harmHygB_R	ATCATTTTAAAGTTCAACATGGTAGTAATCGAATCACCCCCGAGTACCGC**ACAGTTTAGCTTGCCTCGTC**<br>AGTAGTACCTCAGAATCTGTTATCTTCTTGTTCAGTGTTCTATGGAGCCT**TCCAGTATAGCGACCAGCATT**	Amplification of HDR-*HygB* repair template with 50-bp microhomology
FUM1_F(-83up)[1]<br>HygB_R	ATCATCCGACCAAGCCTTCGTAG<br>**TCCAGTATAGCGACCAGCATT**	Amplification of genomic region spanning the RNP1175 recombination site
HygB_F<br>FUM1_R(+114dw)[2]	**ACAGTTTAGCTTGCCTCGTC**<br>AGGGCACATGTACATACGAGTGT	Amplification of genomic region spanning the RNP9269 recombination site
probe5′_koFUM1_F<br>probe3′_koFUM1_R	TTTCCGAGTCCGACACCATG<br>TACGAAGGCTTGGTCGGATG	Amplification of Southern blot probe

Supplementary Information

Full-length gel

Figure 3A

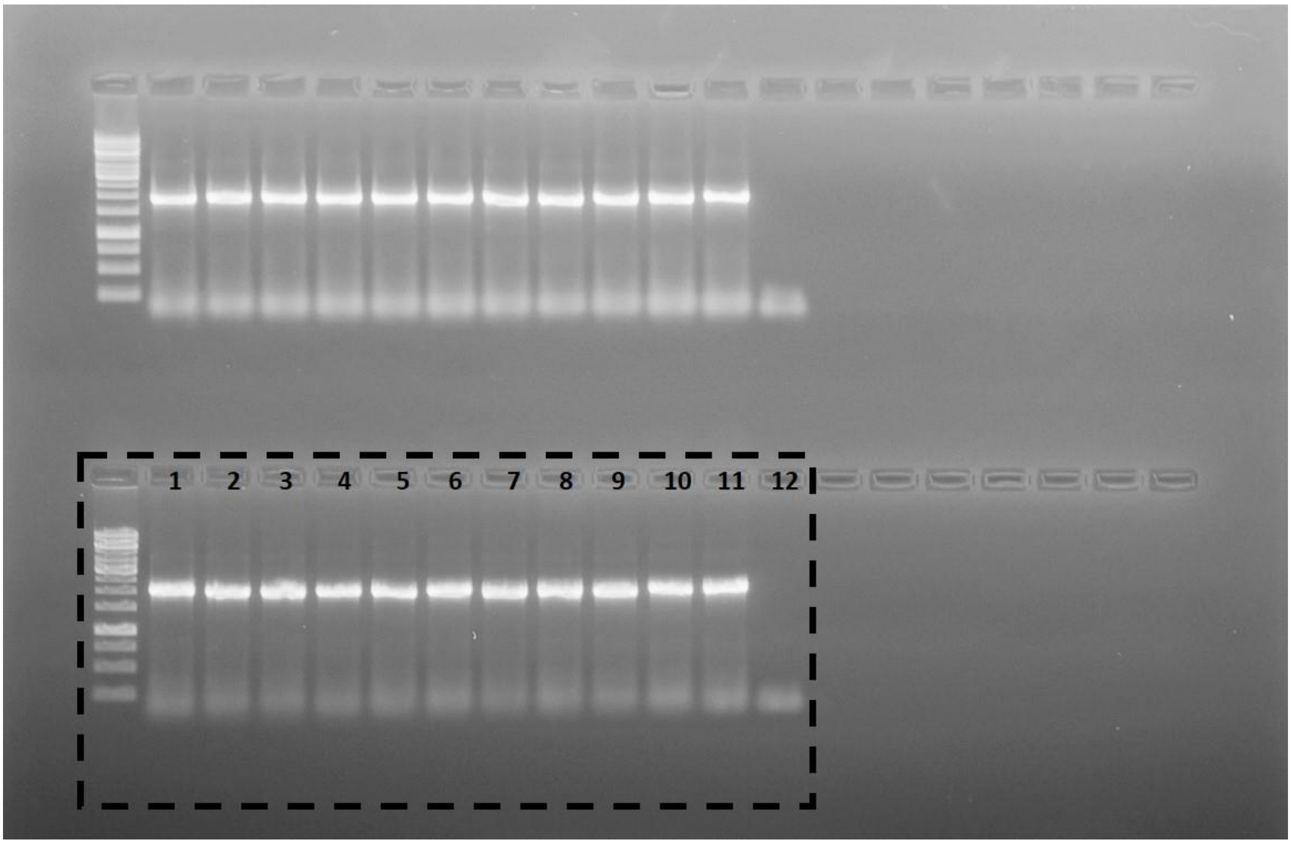

Supplementary Information

Full-length gel

Figure 3B

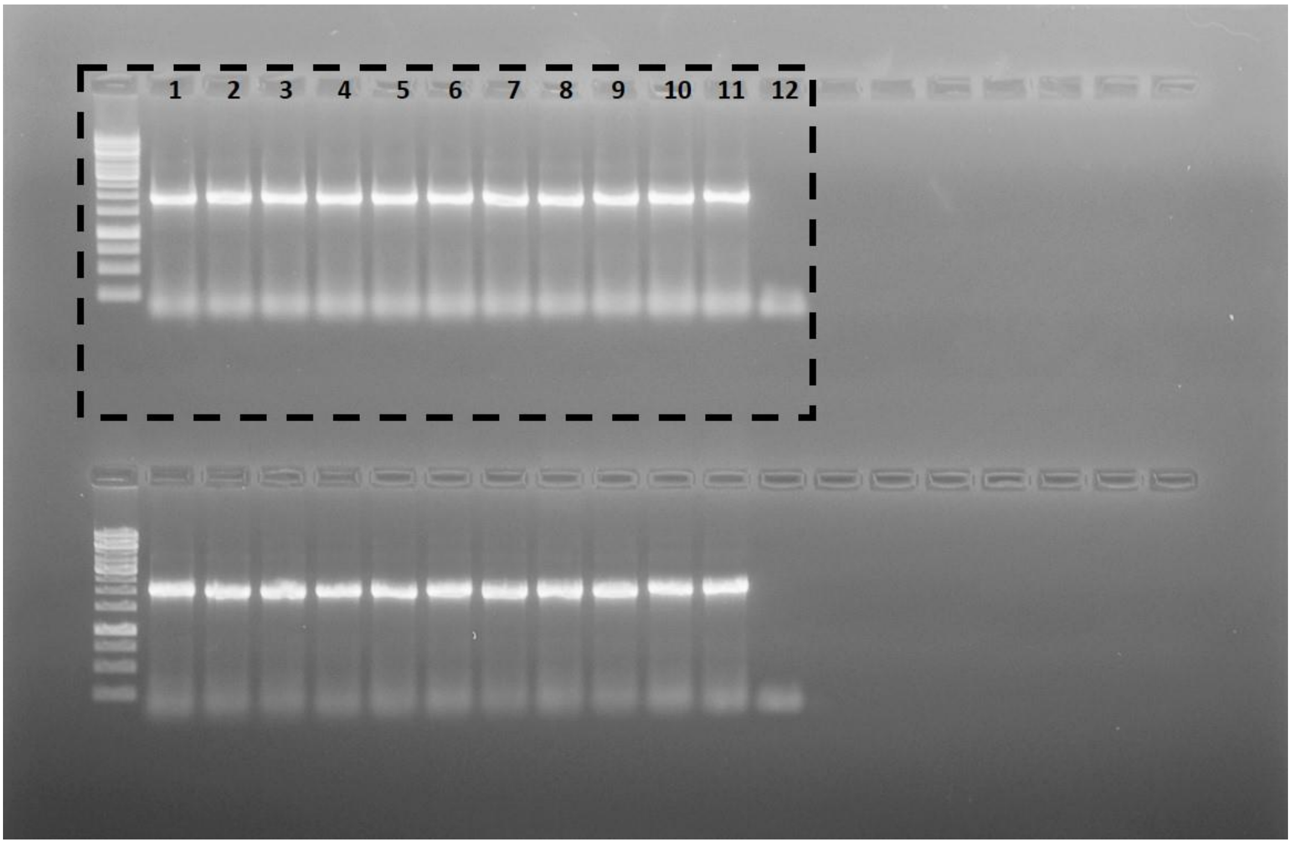

Supplementary Information

Full-length blot

Figure 4

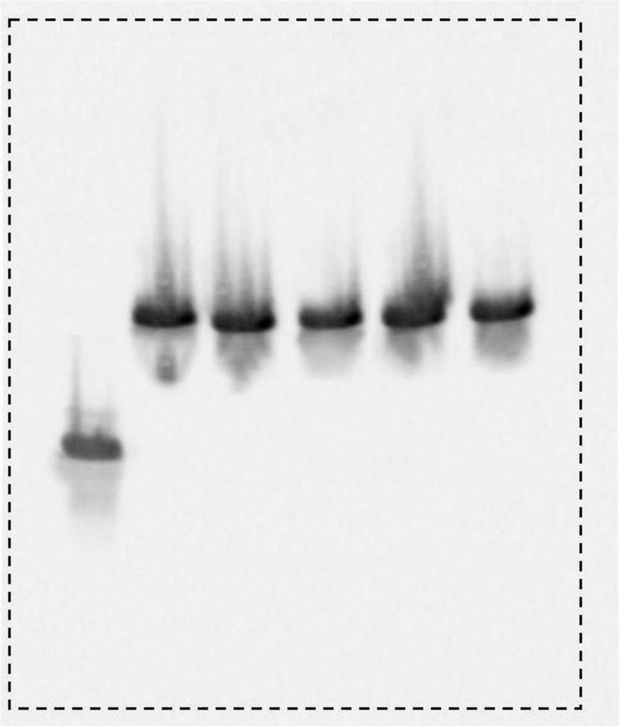

Supplement: Supplementary file 1 — Supplementary Information [file 41598_2019_56270_MOESM1_ESM.pdf]
